# Synergistic Effects of Copper Sites on Apparent Stability of Multicopper Oxidase, Fet3p

**DOI:** 10.3390/ijms19010269

**Published:** 2018-01-16

**Authors:** Erik Sedlák, Gabriel Žoldák, Pernilla Wittung-Stafshede

**Affiliations:** 1 Center for Interdisciplinary Biosciences, Technology and Innovation Park P.J. Šafárik University, Jesenna 5, 041 54 Košice, Slovakia; gabriel.zoldak@upjs.sk; 2 Department of Biology and Biological Engineering, Division of Chemical Biology, Chalmers University of Technology, 412 96 Gothenburg, Sweden

**Keywords:** multicopper oxidases MCO, cupredoxin-like domain, phase diagram method, multidomain protein stability, Fet3p

## Abstract

*Saccharomyces cerevisiae* Fet3p is a multicopper oxidase that contains three cupredoxin-like domains and four copper ions located in three distinct metal sites (T1 in domain 3; T2 and the binuclear T3 at the interface between domains 1 and 3). To probe the role of the copper sites in Fet3p thermodynamic stability, we performed urea-induced unfolding experiments with holo-, apo- and three partially-metallated (T1, T2 and T1/T2 sites depleted of copper) forms of Fet3p. Using a combination of spectroscopic probes (circular dichroism, fluorescence intensity and maximum, 8-anilinonaphthalene-1-sulfonic acid (ANS) emission, oxidase activity and blue color), we reveal that all forms of Fet3p unfold in a four-state reaction with two partially-folded intermediates. Using phase diagrams, it emerged that Fet3p with all copper sites filled had a significantly higher stability as compared to the combined contributions of the individual copper sites. Hence, there is long-range inter-domain communication between distal copper sites that contribute to overall Fet3p stability.

## 1. Introduction

Fet3p from *Saccharomyces cerevisiae* is a multicopper oxidase (MCO) that contains three cupredoxin-like β-barrel domains with four copper ions located in three distinct sites. The T1 copper is coordinated to two histidines and one cysteine and gives rise to Fet3p’s intense blue color (ε_600nm_ ~ 5000 M^−1^·cm^−1^). The other two copper sites, designates T2 and T3, are localized at the interface of the amino (domain 1) and carboxyl (domain 3) terminal domains. The T2 copper ion is coordinated to two histidines, and the third ligand is water or hydroxide. The T3 site contains two copper ions bridged by a non-protein oxygen atom. Each copper in this cluster is coordinated by histidine imidazole rings [1]. The T2 and T3 sites are collectively known as the tri-nuclear cluster (TNC). The ligands for T2 and T3 copper ions are equally distributed between domains 1 and 3, which thus may serve as a structural template for assembly of the TNC.

The MCO family has more than 1000 members sharing the cupredoxin structural motif [2]. Despite a wide array of MCO applications such as biofuel production, organic synthesis, bioremediation and biosensor development [3], very few have been characterized with respect to the role of the metal ions in protein thermal or chemical stability. The effects of copper binding on the stability of small cupredoxin-like proteins (azurin, stellacyanin, plastocyanin, CopC) [4,5,6] have been reported, and most often, removal of copper significantly destabilizes these single-domain proteins. In MCOs, the number of cupredoxin-like domains is increased, and thus, with that, the roles of the copper ions on stability may be more complex. It was shown previously that partial removal of copper ions from the MCO proteins ascorbate oxidase and laccase had only a small stabilizing or slightly destabilizing effect on the folded protein [7,8]. Moreover, a previous functional study of Fet3p and laccase pointed out allosteric coupling between the T1 copper site and TNC [9]. This result supports that copper binding sites in Fet3p communicate, as also suggested from the Fet3p high-resolution structure [1], and predicts possible coupling of copper binding/dissociation with protein folding/unfolding of MCOs.

We have here investigated how the distinct copper ions in Fet3p contribute to overall thermodynamic stability by the use of five forms of Fet3p with different copper sites and the number of copper ions present. Our study of partially-metallated forms of Fet3p thus reveals that the communication of copper binding sites noted earlier on the functional level [9] is also present with respect to structural stability. Because of the complexity of the unfolding transitions, we turned to a phase diagram approach to reveal the involved species and transition midpoints. Our results indicate the presence of allosteric interactions between the copper sites that nonlinearly increase the overall stability of wild-type, Cu-loaded Fet3p.

## 2. Results

### 2.1. Spectral Properties of Folded and Urea-Denatured Wild-Type Fet3p

**Figure 1:**
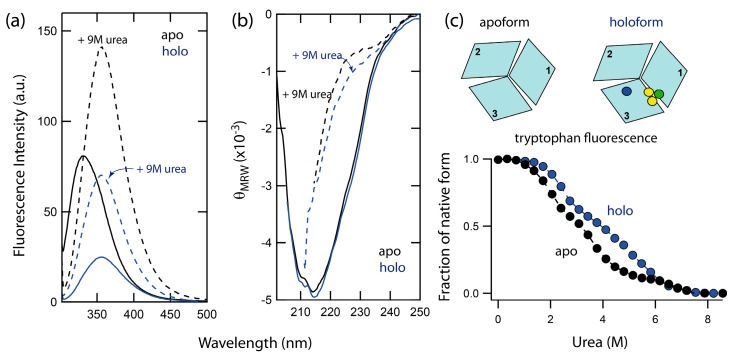
(**a**) Fluorescence of holo (blue lines) and apo (black lines) forms of Fet3p in buffer (solid line) and in the presence of 9 M urea (dashed line). (**b**) Corresponding far-UV CD spectra of holo and apo forms of Fet3p. (**c**) Isothermal urea-induced unfolding of holo (blue circles) and apo Fet3p variants (black circles) probed by Trp fluorescence (ratio F_331nm_/F_355nm_). The circles, in schematically shown structures, indicate locations of T1 (blue), T2 (green), and binuclear T3 (yellow) copper ions. Protein (~5 μM) was measured in 50 mM TrisHCl, pH 7.4 and given urea concentration at 23 °C.

Chemically-induced denaturations of wild-type holo- and apo-forms of Fet3p were accompanied by significant changes in both intrinsic tryptophan fluorescence, as well as the CD signal. In Figure 1, the fluorescence (Fet3p has nine tryptophans) and the far-UV CD (secondary structure) spectra of holo- and apo-forms of Fet3p are shown. The intrinsic tryptophan fluorescence monitors changes in protein tertiary structure via the polarity of the environment near the tryptophan residues. The positions of the fluorescence maxima for holo- and apo-forms of Fet3p are found at the same wavelength, 330 nm, indicating no perturbation of tertiary structure upon metal removal. The increase in fluorescence intensity of the apo-form in comparison with holo-form is likely the result of decreased quenching of fluorescence due to the absence of copper ions. This is in accordance with observations made on other copper-binding proteins with the cupredoxin-like fold such as ceruloplasmin [10] and CopC [6]. The CD spectrum of the holo-form of Fet3p is dominated by β-sheets and minimal α-helical content, which agrees with the reported high-resolution crystal structure [1]. A comparison of the CD spectra of holo- and apo-forms shows no significant changes in the secondary structure of Fet3p due to copper removal. Thus, fluorescence and CD data both indicate similar tertiary and secondary structures of holo- and apo-forms of Fet3p.

### 2.2. Unfolding Reactions of Apo- and Holo-Forms of Fet3p

**Figure 2:**
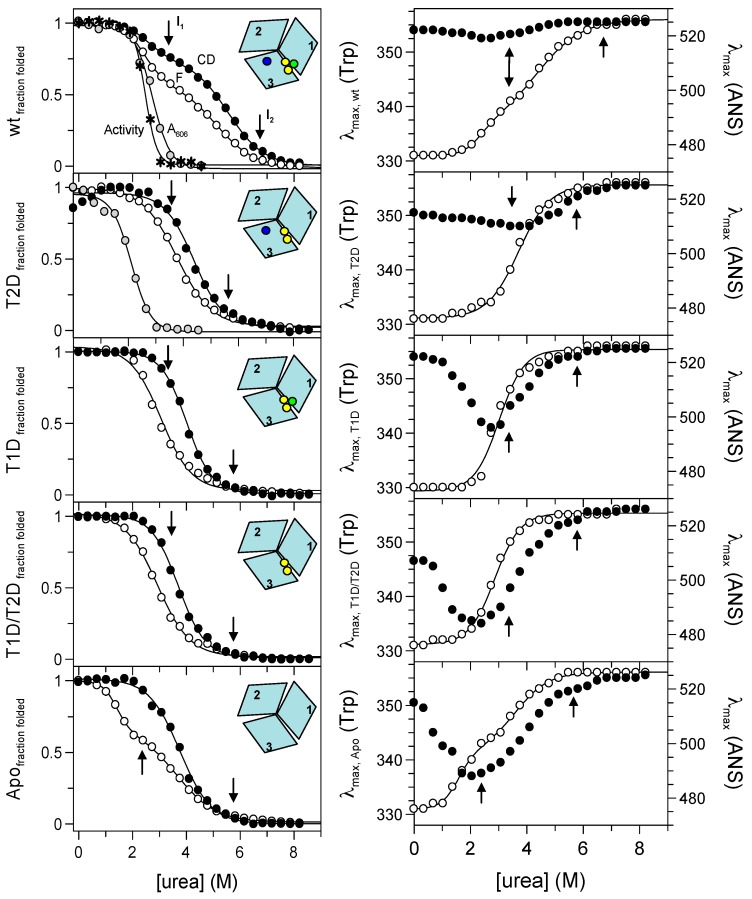
Isothermal unfolding of holo-, T2D, T1D, T1D/T2D and apo-Fet3p variants (**top** to **bottom**) probed by: (**left**) CD at 215 nm (black circles), Trp fluorescence (ratio F_331nm_/F_355nm_; white circles), absorption at 606 nm (grey circles) and oxidase activity (asterisks); (**right**) position of maximum fluorescence emission of Trp (white circles) and 8-anilinonaphthalene-1-sulfonic acid (ANS) (black circles). The arrows indicate the positions of intermediates determined from phase diagram method analysis.

Unfolding of the apo- and holo-form was induced by urea addition at pH 7.4 and 23 °C and monitored by tryptophan fluorescence. The signal changes exhibited a complex dependence on urea concentration, implying a combination of several unfolding steps (Figure 1c). In contrast to a simple two-state reaction, which corresponds to a simple sigmoidal transition, the data for Fet3p reveal the presence of two unfolding intermediates. Importantly, the unfolding curve for holo Fet3p is shifted towards higher urea concentrations as compared to the curve for the apo-form. Thus, the copper ions contribute to the overall protein stability. However, the identities of the unfolding intermediates and the role of individual copper ions cannot be assessed from these data only. Therefore, we turned to several metal-depleted forms of Fet3p and analyzed urea-induced unfolding reactions using a combination of spectroscopic probes: far-UV CD, intrinsic tryptophan fluorescence (intensity and maximum), binding of hydrophobic probe 8-anilinonaphthalene-1-sulfonic acid (ANS), T1 copper site blue color and oxidase activity (Figure 2).

### 2.3. Probing Partially-Metallated Fet3p Variants

#### 2.3.1. Holo-Form of Wild-Type Fet3p

As it follows from far-UV CD and fluorescence signals’ dependencies on urea concentration, holo wild-type Fet3p does not unfold in a two-state process. There is a first transition with a midpoint of ~2.5 M urea, resulting in the loss of about 20–30% of the far-UV CD amplitude and a change in the Trp emission. For analysis of Trp fluorescence changes, we used the ratio of emission amplitude at 331 nm and 355 nm. In general, the ratio F_331nm_/F_355nm_ provides a better reporter (less noise) than the observed signals at individual wavelengths. However, the use of the ratio of spectroscopic signals need to be taken with caution as recently reported [11]. In all our cases, using the fluorescence ratio method gave less than a 2% difference as compared to the analysis of the individual signals.

The first unfolding step with a midpoint at ~2.5 M urea is followed by a second transition with a midpoint at ~5.3 M urea. According to the far-UV CD and the Trp emission maximum, in the second transition, the protein attained the fully-unfolded form. In the case of the holo-form of wild-type Fet3p, the effect of urea on the environment of the T1 copper ion can be monitored by visible absorbance at 606 nm and by oxidase activity (Figure 2). The blue color, as well as oxidation of aromatic diamine substrates require an intact T1 copper site. We find inactivation of Fet3p, as well as a loss of 606-nm absorbance to take place in transitions with urea midpoints at ~2.7 M and ~2.5 M urea, respectively. These observations strongly indicate that perturbation of the T1 copper ion coincides with the first unfolding transition observed by fluorescence and CD.

The unfolding reaction of holo Fet3p was also probed via changes in the position of the emission maxima of Trp and ANS fluorescence signals (Figure 2). The Trp emission maximum is a sensitive probe of polarity and dynamics of the environment near Trp residues [12]. Similarly, the ANS emission depends on its close environment, and it is often used to detect intermediate species as it does not significantly bind to either folded or unfolded protein forms [13]. The dependence of the Trp emission maximum on urea concentration indicates again that holo Fet3p unfolding proceeds in at least two steps, supporting the above results from CD and fluorescence intensity measurements. The Trp maximum changes are accompanied with only minimal changes in ANS emission maximum for holo-Fet3p, which indicates the absence of intermediates with exposed hydrophobic patches.

It needs to be stressed that neither of the unfolding steps is reversible as the ‘refolded state’ of Fet3p (dilution into buffer from 9 M urea) is characterized by increased negative CD signal at 210 nm (indicating increased β-sheet content), a Trp emission maximum at ~348 nm (indicating perturbed tertiary structure) and a higher fluorescence intensity than that of the native state (Figure S1). This is similar to previously published observations on attempts to refold human ceruloplasmin [10]. The irreversibility of the transitions prevents detailed thermodynamic analysis. Therefore, for comparison purposes only, apparent values of all unfolding transition midpoints, [urea]_1/2_, detected with the different methods are listed in Table S1. 

#### 2.3.2. T2D Variant of Fet3p

Elimination of the type 2 copper (at the interface between domains 1 and 3), due to H18 Q mutation in the copper binding site (refer to the Materials and Methods), results in a protein that exhibits an apparent one-step unfolding transition monitored by: (i) the absorbance at 606 nm (midpoint at ~2.1 M urea) and (ii) fluorescence and CD ([urea]_1/2_ of ~3.8 M and ~4.4 M, respectively). Because the apparent one-step unfolding transitions are observed at different [urea]_1/2_ depending on the detection method, this clearly suggests the presence of intermediate(s) during unfolding. Urea-induced effects on the Trp emission maximum are also characterized by an apparent one-step transition with a [urea]_1/2_ ~ 3.7 M. The ANS emission maximum depends on urea in a more pronounced way than in the case of holo wild-type Fet3p.

#### 2.3.3. T1D Variant of Fet3p

A substitution of cysteine 484 to serine results in the loss of T1 copper ion (in domain 3), and it is found to destabilize the protein more than the elimination of the T2 copper. This is indicated by the midpoints of urea-induced transitions of fluorescence and CD observed at ~3.1 M and ~4.0 M urea, respectively. Accordingly, the urea-induced transition monitored by Trp emission maximum is also shifted to lower urea concentrations with [urea]_1/2_ ~3.0 M. The dependence of the ANS emission maximum wavelength shows a significant minimum at ~3.0 M urea, which thus correlates with the midpoint of transitions probed by Trp intensity and maximum position.

#### 2.3.4. T1D/T2D Variant of Fet3p

The double mutant of Fet3p H81Q/C484S, with absent copper ions of both type 1 and type 2 copper sites, is destabilized more than the individual T1D and T2D variants of the Fet3p. This protein variant unfolds in an apparent one-step transition as monitored by fluorescence and CD with [urea]_1/2_ ~2.9 M and ~3.7 M, respectively. A one-step transition with a midpoint at ~2.9 M urea is also observed when following the Trp emission changes, and the ANS emission maximum is characterized by a pronounced minimum at ~2.4 M urea.

#### 2.3.5. Apo-Form of Wild-Type Fet3p

We probed unfolding reactions of the apo-forms of all Fet3p variants. Because the observed transitions were similar for all apo-forms, for clarity, we show only the results for the apo-form of wild-type Fet3p. The absence of all copper ions in the Fet3p structure leads to a re-appearance of an apparent two-step transition (like the wild-type holo-form) when monitored by fluorescence with midpoints at ~1.4 M and 3.7 M urea. Similarly, the dependence of the Trp emission maximum wavelength on urea also has a two-step transition character with [urea]_1/2_ ~1.6 M and 3.6 M. On the other hand, the urea-induced transition probed by CD is a one-step process with a midpoint at ~3.7 M urea. Again, the ANS emission maximum depends on the urea concentration in a complex way with a minimum at ~2 M urea.

#### 2.3.6. Phase Diagram Analysis of Fet3p Variant Data

**Figure 3:**
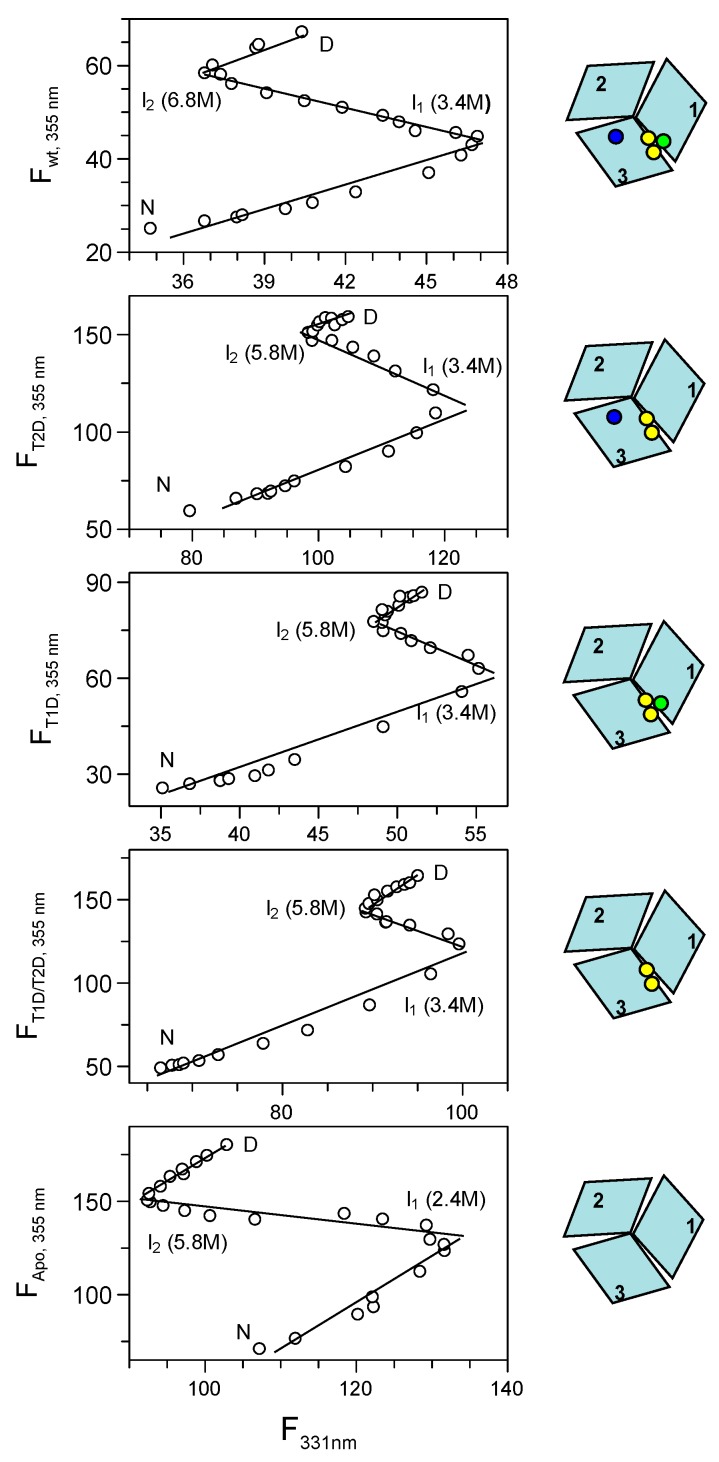
Phase diagram method analysis of holo-, T2D, T1D, T1D/T2D and apo-Fet3p variants (top to bottom) using fluorescence intensities at 331 nm and 355 nm (upon excitation at 295 nm) as the two extensive variables. All phase diagrams indicate the population of two intermediates, which are highly populated at the indicated concentrations of urea.

Because of the complexity of the urea-induced unfolding reactions of the various Fet3p variants, the data were analyzed by the more powerful phase diagram method [14,15]. The basis of this method is a pairwise correlation of two different extensive parameters (two different detection probes), such as fluorescence intensities at different wavelengths or fluorescence intensity and CD signal, but not signal ratios or the position of fluorescence maxima (which are not parameters that depend on a protein fraction in the additive manner). This approach has the ability to reveal hidden intermediates not easily detected by normal data fitting. For a two-state transition, a phase diagram plot should be linear, and any nonlinearity will correspond to deviations from a two-state transition. The number of linear portions in a plot, *n*, indicates *n* + 1 species present in the reaction. It is important that the phase diagram method is applied to sets of data measured under identical conditions. For our sets of data, we plot the Trp fluorescence intensity values at 331 and 355 nm against each other (Figure 3). However, any two parameters can be used, and for example, plotting of fluorescence at 331 or 355 nm versus the CD signal at 215 nm led to similar results (Figure S2). Inspection of the plots in Figure 3 shows that the diagrams are not linear in any case, but instead, the data indicate the presence of unfolding intermediates. Because there are three linear regions in all phase diagrams, this implies that all species unfold in reactions involving four species, of which one is folded and one is the fully unfolded state and the remaining two are unfolding intermediates. The phase diagram methods estimate conditions, i.e., in our case urea concentrations, at which the individual states/intermediates are populated. These conditions can be identified at the break points between linear regions in a model independent way, and hence, these values are considered reliable for analysis.

Upon closer examination of the phase diagrams, we find that the first intermediate in all Fet3p variants (except apo Fet3p) is populated at ~3.4 M urea. For the apo-form of Fet3p, the first intermediate populates already at lower urea, ~2.4 M urea. The second intermediate of holo wild-type Fet3p occurs at ~6.8 M urea, whereas for the other variants, the second intermediate is populated at ~5.8 M urea. At even higher urea concentrations, the second intermediate converts to the fully-unfolded state.

The urea concentrations for the maximal population of the first and second intermediates, as revealed by the phase diagrams, are indicated in Figure 2 by arrows for each specific Fet3p variant. The first and the second unfolding transitions for holo- and apo-forms of Fet3p are clearly distinguishable and appear to correspond to the formation and unfolding of the first intermediate, as detected in the phase diagram. For the partially-metallated forms of Fet3p, the situation is more complex as the observed dependencies from individual probes (CD and fluorescence) do not reveal the presence of intermediate(s). Furthermore, in all cases, the third transition (from the second intermediate to the unfolded state) is detected neither by CD nor Trp fluorescence. However, there is an indication of a third transition at high urea concentrations in the ANS fluorescence data, particularly for the apo-form of Fet3p (Figure 2).

## 3. Discussion

We previously showed the existence of a trade-off between protein thermal stability and metal binding in Fet3p [16]. Similar to the Fet3p thermal data, partial removal of copper ions from other MCOs, such as ascorbate oxidase or laccase, did not destabilize the overall structure, and in some cases, it even increased protein stability [7,8]. To obtain a better understanding of the role of the copper ions in MCO proteins, we here investigated the role of the copper ions in the chemically-induced unfolding reaction of Fet3p using holo-, apo- and three partially-metallated forms. Our current findings support previous observations that partial removal of copper ions can stabilize certain forms of Fet3p towards thermal denaturation [16]. For example, removal of the T2 copper changes an apparent two-step transition of holo wild-type Fet3p, with midpoints at ~2.6 M and ~5.5 M urea, to an apparent one-step transition for T2D Fet3p with a single midpoint at ~4.4 M urea as probed by the far-UV CD.

However, quantitative analysis of the isothermal (chemical) denaturation data of Fet3p and its variants is complicated because of the existence of several partially overlapping urea-induced transitions, which are difficult to distinguish. In fact, in the partially-metallated forms of Fet3p, both CD and fluorescence probes suggest one-step transitions, but because they take place with different urea dependencies, this indicates the presence of intermediate(s). Thus, for more reliable analysis, we utilized the phase diagram method [14,15]. Applying this method to the unfolding data at hand revealed the presence of allosteric effects of the copper ions on Fet3p stability and cooperativity.

**Figure 4:**
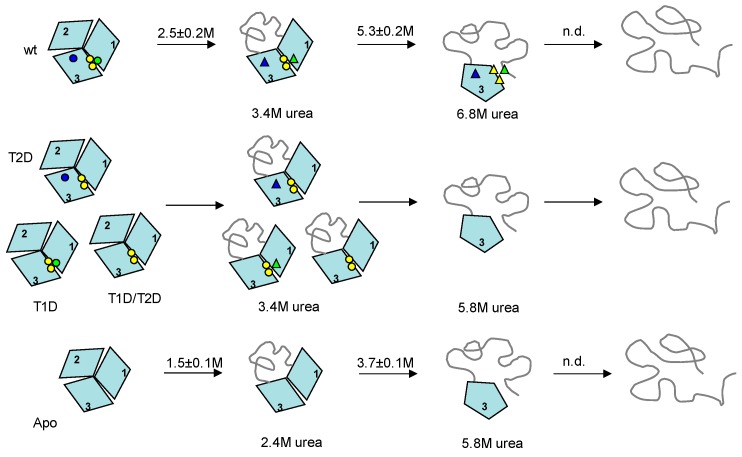
Unfolding pathways of wild-type (**upper** scheme), T2D, T1D, T1D/T2D (**center** scheme) and apo- (**bottom** scheme) Fet3p variants. The apparent transition midpoints (average values from CD and fluorescence measurements, Table S1) for holo- and apo-forms of Fet3p are shown above the arrows (the midpoints for partially-metallated forms were not identified by the individual measurements). Native-like coordination of copper ions is shown as circles, and perturbed copper coordination (but still bound copper) is shown as triangles. The circles and the triangles, in schematically shown structures, indicate locations of T1 (blue), T2 (green), and binuclear T3 (yellow) copper ions.

Our conclusions (discussed below) with respect to unfolding steps and the characteristics of the intermediates formed are schematically summarized in Figure 4. From the phase diagram analysis, holo-Fet3p unfolds through two intermediates populated at ~3.4 M and 6.8 M urea. All partially-metallated Fet3p forms also unfold through two intermediates populated at ~3.4 M and 5.8 M urea. For apo-Fet3p, the two intermediates are populated at ~2.4 and ~5.8 M urea. Based on the facts that (i) all cupredoxin domains are structurally similar, (ii) only domain 2 lacks a copper site, (iii) earlier thermal denaturation of Fet3p indicated that domain 2 is the most instable domain [16], and (iv) by analogy to observations on the homologous ceruloplasmin showing the N- and C-terminal domains connected with the TNC site to be the most stable part [10], we suggest that the first transition corresponds to unfolding of Fet3p domain 2. Since this transition is accompanied by the loss of oxidase activity (again, matching the results for ceruloplasmin [10]), as well as the disappearance of blue color, it suggests that unfolding of domain 2 affects domain 3 such that the T1 copper coordination is perturbed (but the copper may still be coordinated).

Because apo- and partially-metallated forms all show the presence of an intermediate state at ~5.8 M urea, this intermediate cannot be stabilized by the individual copper ions that vary among these species. On the other hand, the population of the first intermediate is shifted from ~2.4 M urea for the apo-form to ~3.4 M urea in fully- or partially-metallated forms of Fet3p. Here, it is reasonable to suggest that the presence of the T3 copper ions (present in all partially-metallated forms) at the interface between domains 1 and 3 stabilizes the first intermediate. The fact that the second intermediate of the holo wild-type Fet3p is populated at higher urea concentration, ~6.8 M, than the second intermediate of all partially-metallated forms, ~5.8 M, indicates that both T1 and T2 sites are needed to obtain the higher stability of the first intermediate (and delay formation of the second intermediate). Removal of either T1 or T2 copper ions leads to a destabilization of the first intermediate that is the same if one of the sites or both sites are removed. Because domain 3 involves ligands to all three copper sites and thereby has many stabilizing interactions, it appears most likely that the second transition involves unfolding of domain 1 and not domain 3 [1]. Thus, the combination of T1 and T2 copper ions (but not T1 and T2 in isolation) facilitates a stabilization of domain 1 in the first intermediate. This is also evident by the ANS data that suggest more ANS binding and thus more exposed surfaces, in the first intermediate of all variants lacking one or more copper, as compared to for the same in the holo wild-type case.

The coppers in T1 and T2 are about 15 Å apart, and hence, a direct interaction or mediated through a single side-chain is excluded; instead, we propose that interactions are transmitted by the secondary structure elements. The copper ions in T2 and T3 centers in Fet3p are coordinated by domains 1 and 3 by utilizing local, adjacent residues, as well as non-local residues. In contrast, copper in T1 is coordinated solely by domain 3 residues, and hence, the contribution to the stability of other domains has to be transmitted through interactions through secondary structure elements. To analyze how putative interacting residues are conserved among homologs of Fet3p, we performed sequence alignment using CLUSTALW (Figure S3). From this, we could identify several conserved residues and sequence motifs, including the residues responsible for the coordination of copper ions. In particular, His81 (T2), His83 (T3), His128 (T3) and His489 (T1) were found to be conserved among homolog proteins. Several conserved residues are organized in the form of sequence motifs for which the role remains unclear. Long-range interactions between T1 and T2 copper ions may be facilitated by residues His413 and His416 in the same β-strand. The communication between T1 and TNC copper sites may be further strengthened by coordination of the T1 copper to Cys484 and one of the T3 copper ions via His485 in domain 3 and, at the same time, via His83 in the same β-strand as a T2 ligand (His85) in domain 1 (Figure S3). An allosteric interaction between T1 and TNC copper sites was recently recognized by spectroscopic studies revealing that perturbation of the T3 site alters the redox potential of the T1 copper ion [9]. It was also shown that in homologous blue oxidases, not only structural, but also dynamic properties are conserved, as demonstrated by the analysis of extremophilic enzymes, and they appeared to be a pre-requisite for copper binding and function [17,18]. Together with our results, one may propose that networks of interactions play combined roles in the function, dynamics and stability of Fet3p.

The unfolding schemes for the Fet3p variants (Figure 4) give implications for plausible refolding pathways. One may simply imagine a reversal of the unfolding path. The last domain to unfold, domain 3, is likely the first domain to refold. This can take place in the absence of metals, and domain 3 provides the starting scaffold for subsequent copper binding. As soon as domain 3 is folded, copper ions can start to bind, although true copper sites are not yet formed. The transition from the intermediate with domain 3 folded to the next, more folded, intermediate occurs at the same urea concentration for all partially-metallated and the apo-forms of Fet3p, but for the wild-type protein, this transition is shifted to higher urea concentrations. Thus, the second refolding step likely involves folding of domain 1 and correct coordination of copper ions at the interface of domains 1 and 3, i.e., formation of the tri-nuclear center (TNC). Furthermore, T2 and T1 copper will be in place in this intermediate, although at least the T1 copper is not yet correctly arranged to give rise to blue color. The final step in refolding would then be folding of the least-stable (metal-site free) domain 2 together with correct coordination of copper in T1 in domain 1. We propose that to assure correct (and early) delivery of copper to Fet3p during it various folding steps in the secretory path in vivo, particular chaperones, specific biosynthetic conditions and/or direct interaction with the copper delivering ATPase CCC2, appear necessary. 

In conclusion, using a phase diagram approach in combination with urea-induced unfolding experiments of various Fet3p variants, we have discovered that the copper sites in Fet3p provide additional protein stability by long-range coupling. In addition, we provide a tentative three-step pathway of domain folding and metal insertion events for Fet3p that may be extended to other proteins in the MCO family.

## 4. Materials and Methods

### 4.1. Protein Preparation

The holo-forms of wild-type and mutant Fet3p variants were prepared as described [19,20]. The studied variants are as follows: T1D contains a Cys-484 to Ser mutation at the T1 site; T2D contains a His-81 to Gln mutation at the T2 site; T1D/T2D is a double mutant, which contains both the above changes [19]. The apo-form of wild-type Fet3p, as well as of all partially-metallated Fet3p variants, were made by initial dialysis at 4 °C in 50 mM ascorbate and 0.1 M TrisHCl, pH 7.2. The second step was dialysis against the same Tris buffer including 50 mM NaCN and 10 mM EDTA. The final step was dialysis into 50 mM phosphate buffer, pH 7 (the condition used in all subsequent experiments). The apo-forms of all Fet3p variants prepared this way contained <0.5 copper per protein determined by the method of Felsenfeld [21]. The metal contents of the Cu-loaded forms of holo wild-type Fet3p and the three Fet3p variants were determined by flameless atomic absorption spectrophotometry. Contamination of Fe and Zn was below the detection limit in all proteins (i.e., <0.1 atoms per protein molecule). The Cu analyses showed that the holo-form of wild-type Fet3p has 3.8 ± 0.2 Cu atoms per protein molecule; both T1D and T2D Fet3p variants have 2.7 ± 0.3 Cu atoms per protein molecule; and the T1D/T2D variant has 1.8 ± 0.2 Cu atoms per Fet3p molecule. The Fet3p concentration, for the purpose of the copper quantification, was determined by the Bradford assay on protein samples dialyzed against Chelex-treated buffer [19].

### 4.2. Isothermal Unfolding Experiments

Samples for unfolding were prepared by suitable mixing of Tris-HCl and urea solution (9.3 M urea in 50 mM TrisHCl, pH 7.4) and protein (protein solution in Tris-HCl) with a final protein concentration of ~5 μM. Samples were incubated for 1 h at 23 °C. For achieving a better signal/noise ratio, the far UV circular dichroism (CD) was obtained from the averaged signal at 215 nm collected for 120 s. The same samples were used for fluorescence measurements. Tryptophan fluorescence spectra were obtained after excitation at 295 nm. The 8-anilinonaphthalene-1-sulfonic acid (ANS) fluorescence was obtained from protein/urea samples including 200 μM ANS (excitation at 390 nm). In all fluorescence measurements, both the intensity and position of emission maximum were determined from spectra obtained by averaging five consecutive scans.

### 4.3. Spectroscopy

Far-UV CD spectra and isothermal denaturation experiments were recorded on a Jasco-810 (1-mm path). For ANS fluorescence, protein was mixed with 200 μM ANS and incubated for 1 h before experiments. Tryptophan and ANS fluorescence signals at 335 nm (excitation at 295 nm) and 510 nm (excitation at 390 nm), respectively, were monitored on a Varian Eclipse (1 cm × 1 cm cell). Absorption at 330 nm and 510 nm was monitored on a Cary-50 (1-cm path). All measurements were performed in duplicate.

### 4.4. Activity Assay

Oxidase activity of wild-type holo Fet3p was tested with o-dianisidine as a substrate [22]. Activity as a function of urea concentration was determined as follows: a sample was incubated at a given urea concentration for 1 h at 23 °C; then, a 25 μL of aliquot of the solution was transferred into 100 μL of 100 mM acetate, pH 5.0. The oxidase activity was then measured at 23 °C in duplicate. 

### 4.5. Data Analysis

The CD and fluorescence data were fit to 2-step or 3-step reactions according to the equation for the analysis of isothermal denaturation as described previously [23,24].

## Supplementary Materials

The following are available online at www.mdpi.com/1422-0067/19/1/269/s1.
