# Supplementary material for: Synergistic Effects of Copper Sites on Apparent Stability of Multicopper Oxidase, Fet3p
Source: Int J Mol Sci. 2018 Jan 16;19(1):269. doi: 10.3390/ijms19010269 (PMC5796215; doi:10.3390/ijms19010269)
Supplement: Supplementary file 1 [file ijms-19-00269-s001.pdf]

Supplementary Material

Synergistic effects of copper sites on apparent stability of multicopper oxidase, Fet3p

Erik Sedlák, Gabriel Žoldák and Pernilla Wittung-Stafshede

Table S1. Midpoints, $[urea]_{1/2}$, of urea-induced transitions of all studied forms of Fet3p measured by ellipticity at 215 nm, Trp fluorescence intensity (ratio F_{331nm}/F_{355nm}), position of maximum emission of Trp fluorescence, absorbance at 606 nm, and oxidase activity (in the case of holo Fet3p). The uncertainty in determination of values $[urea]_{1/2}$ is ±0.1M urea.

	**$[urea]_{1/2}$ (M)**				
Fet3p form	CD_{215nm}	F_{331nm}/F_{355nm}	F_{Trp}(nm)	A_{606nm}	activity
wt	2.6/5.5	2.4/5.1	2.3/4.4	2.7	2.5
T2D	4.4	3.8	3.7	2.1	n.a.
T1D	4.0	3.1	3.0	n.a.	n.a.
T1D/T2D	3.7	2.9	2.9	n.a.	n.a.
apo	3.8	1.4/3.7	1.6/3.6	n.a.	n.a.

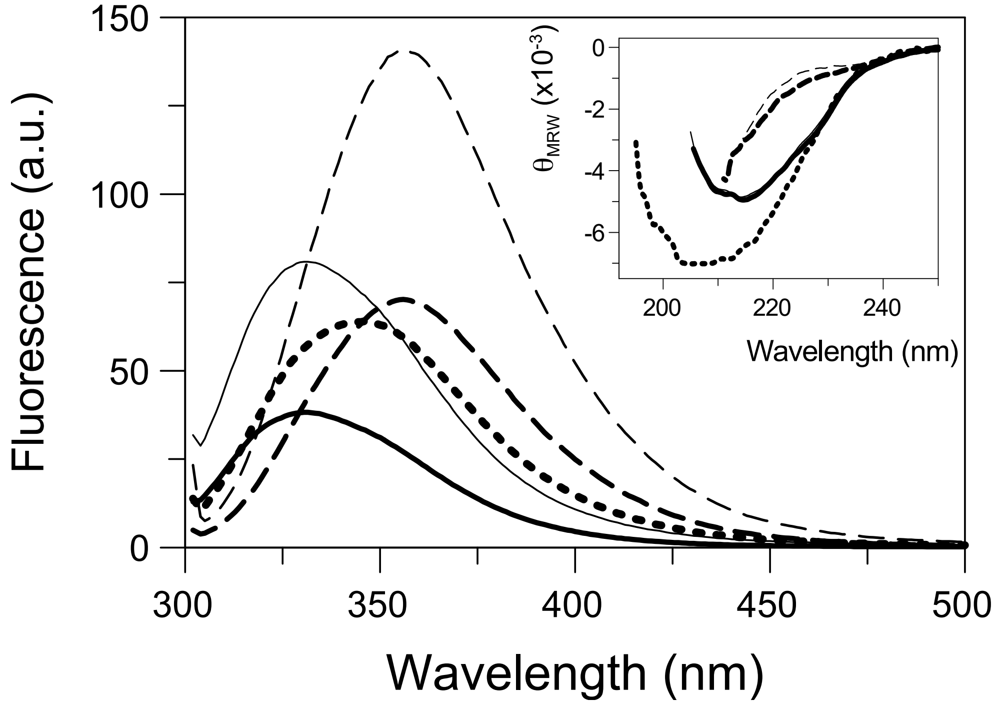

Figure S1. Fluorescence of holo (thick line) and apo (thin line) forms of Fet3p in buffer (solid line) and in the presence of 9M urea (dashed line). Fluorescence of the refolded form of holo-Fet3p is shown in dotted line. Inset: Corresponding far-UV CD spectra of holo and apo forms of Fet3p.

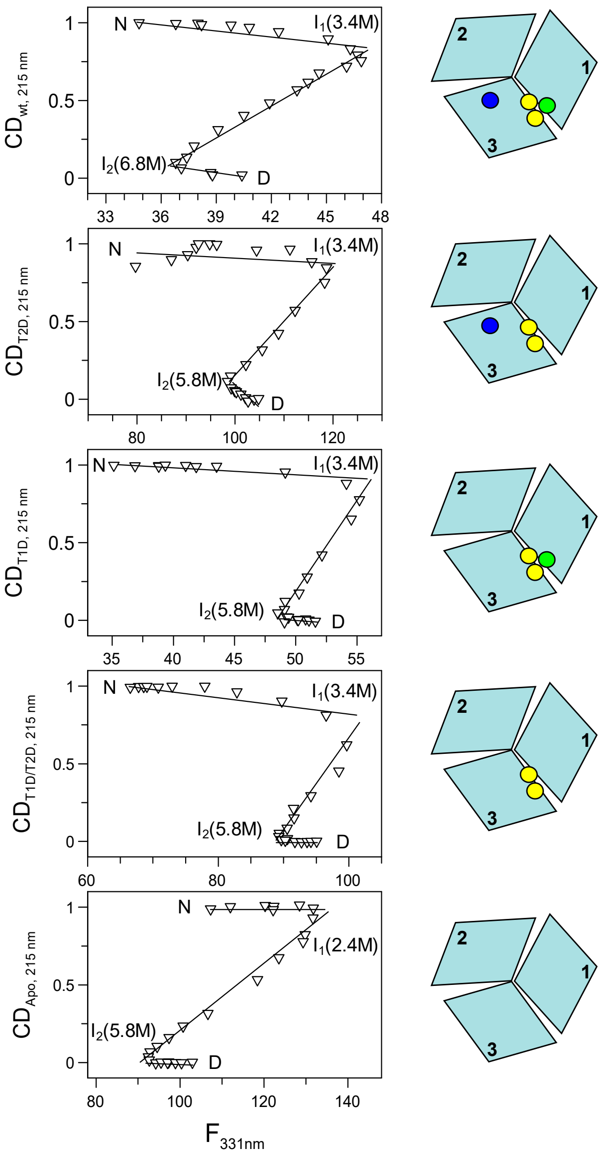

Figure S2. Phase diagram method analysis of holo, T2D, T1D, T1D/T2D and apo Fet3p variants (top to bottom) using fluorescence intensities at 331 nm (upon excitation at 295 nm) and (normalized) ellipticity at 215 nm as the two extensive variables. All phase diagrams indicates the population of two intermediates, which are highly populated at the indicated concentrations of urea.

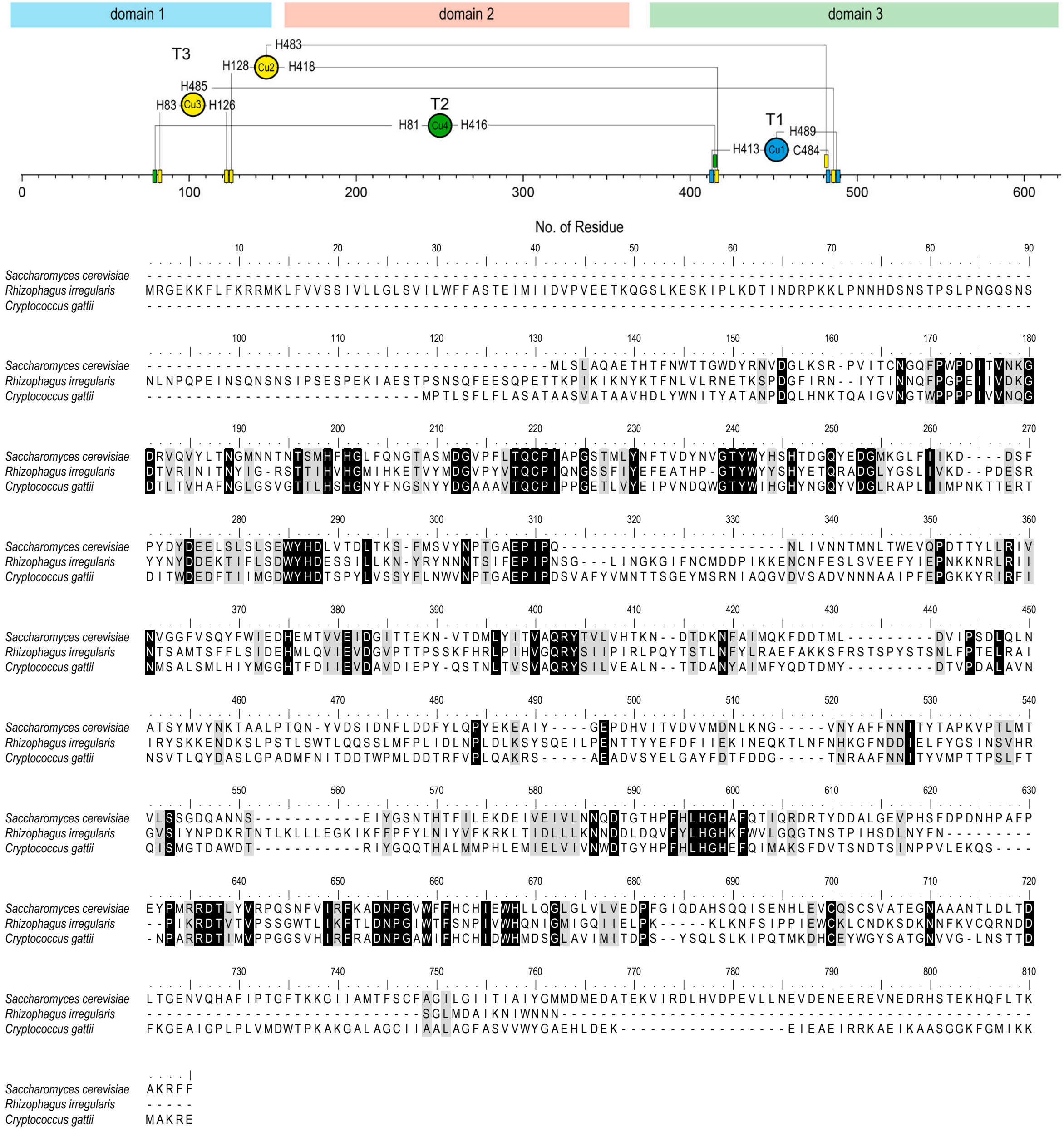

Figure S3. The coordination of copper ions by Fet3p residues, their localization within the domains and their interconnection are shown schematically in the upper part and the sequence alignment of Fet3p homologous proteins (the lower part) shows the conservative (black boxes) and similar (grey boxes) residues.
